# Supplementary material for: Cytomegalovirus vaccine vector-induced effector memory CD4 + T cells protect cynomolgus macaques from lethal aerosolized heterologous avian influenza challenge
Source: Nat Commun. 2024 Jul 19;15:6007. doi: 10.1038/s41467-024-50345-6 (PMC11272155; doi:10.1038/s41467-024-50345-6)
Supplement: Supplementary file 1 — Supplementary Information [file 41467_2024_50345_MOESM1_ESM.pdf]

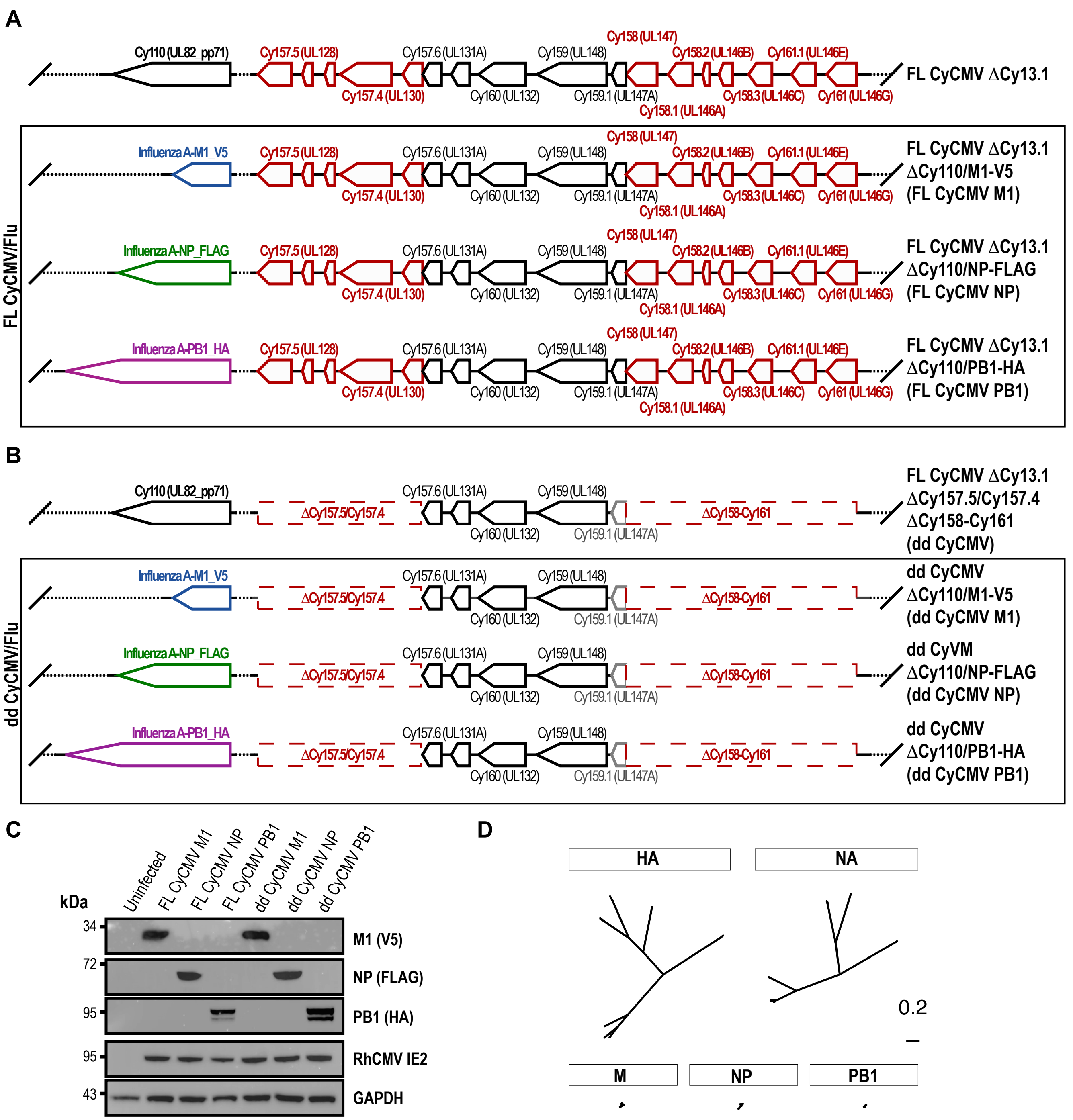

Supplementary figure 1: Genetic configuration of CyCMV vectors utilized. (A) Schematic representation of the full length (FL) CyCMV vector is shown at top. Note that in all constructs the Cy13.1 gene is deleted to facilitate growth in vitro. The box below depicts the three FL CyCMV-derived vectors (cumulatively named FL CyCMV/Flu) where Cy110, the orthologue of HCMV UL82 encoding the pp71 protein, is replaced by the coding sequence for either influenza M1 with a V5 tag, NP with a FLAG tag, or PB1 with an HA tag, as indicated. Pointed boxes indicate distinct exons. **(B)** Schematic representation of the same genomic regions shown in panel A, but for "double deleted" (dd) CyCMV vectors, i.e. deleted for the gene regions Cy157.5-Cy157.4, and Cy158-Cy161. The parental vector is shown at the top with red boxes indicating genes deleted from FL CyCMV. The box below depicts the three dd CyCMV-derived vectors (cumulatively named dd CyCMV/Flu) where the pp71-encoding Cy110 gene is replaced by the coding sequences for either influenza M1-V5, N-FLAG tag, or PB1-HA. **(C)** Immunoblots confirming expression of the influenza transgenes in primary rhesus fibroblasts infected with one of each of the three FL or dd CyCMV/Flu vaccine vectors at an MOI of 3. The epitope tags recognized by specific antibodies to detect each transgene is listed on the right. **(D)** Phylogenetic trees of influenza virus hemagglutinin (HA), neuraminidase (NA), M1, NP, or PB1 sequences representing viral isolates from 1918-2020.

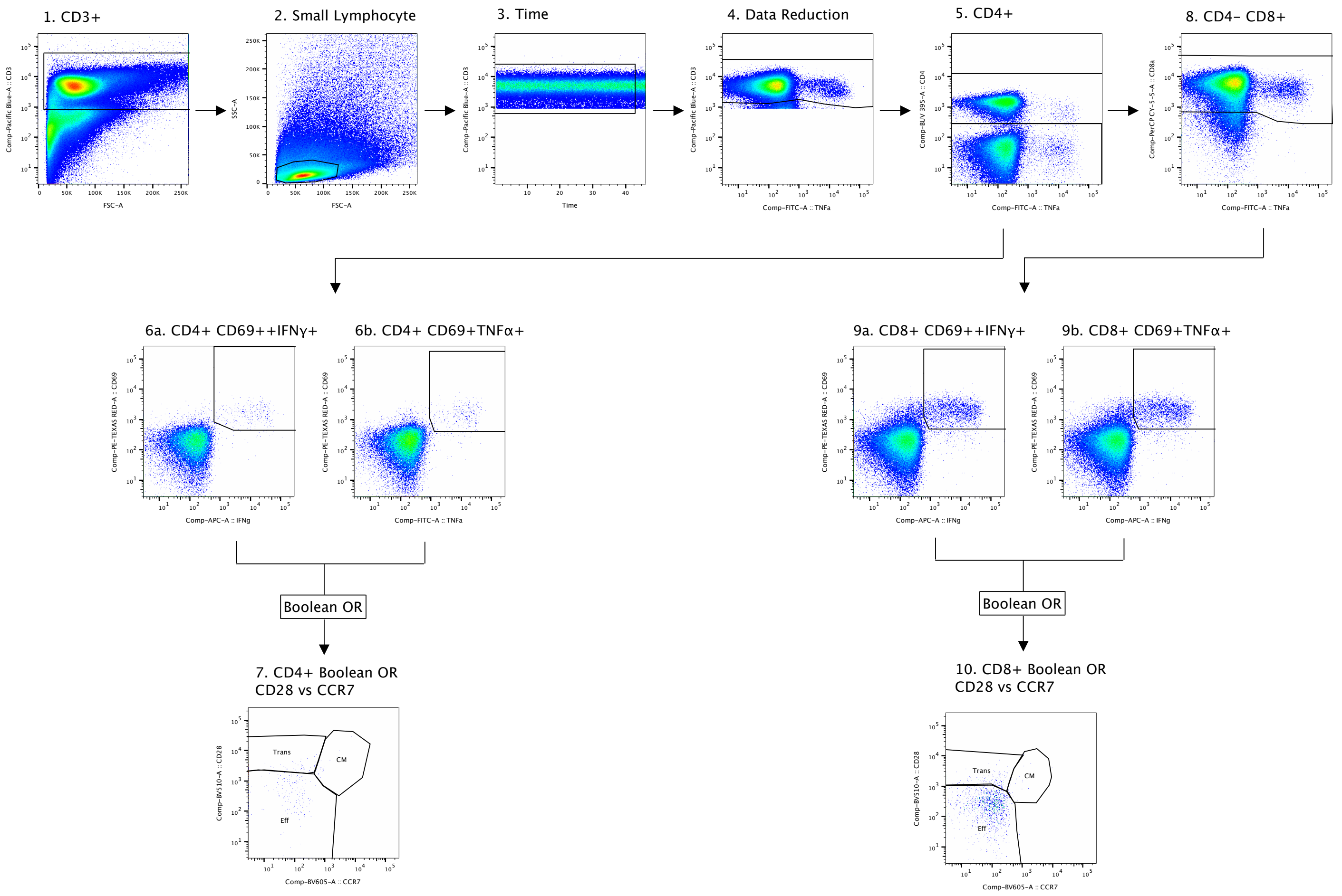

Supplementary figure 2: Representative flow data and gating strategy. Shown are representative flow plots and gating strategies used in the study. The specific plots here are shown for PBMC from animal PA64 following stimulation with NP peptides for analysis of memory phenotype.

ddCyCMV/flu-vaccinated MCM				
**MCM ID**	**Source (vendor)**	**Sex**	**Age (years)**	**Influenza A/B seropositivity**
PA62	Prelabs, LLC	Male	3.73	-
PA63	Prelabs, LLC	Male	3.71	-
PA70	Prelabs, LLC	Female	3.72	-
PA71	Prelabs, LLC	Female	3.65	-
PA72	Prelabs, LLC	Female	3.69	-
PA77	Prelabs, LLC	Male	3.7	-
**FL CyCMV/flu-vaccinated MCM**				
**MCM ID**	**Vendor**	**Sex**	**Age**	**Influenza A/B seropositivity**
PA64	Prelabs, LLC	Female	3.72	-
PA65	Prelabs, LLC	Female	3.61	-
PA67	Prelabs, LLC	Male	3.72	-
PA69	Prelabs, LLC	Male	3.78	-
PA73	Prelabs, LLC	Female	3.64	-
PA75	Prelabs, LLC	Male	3.72	-
**Unvaccinated control MCM**				
**MCM ID**	**Vendor**	**Sex**	**Age**	**Influenza A/B seropositivity**
PA66	Prelabs, LLC	Female	3.62	-
PA68	Prelabs, LLC	Female	3.93	-
PA74	Prelabs, LLC	Female	3.75	-
PA76	Prelabs, LLC	Male	3.69	-
PA78	Prelabs, LLC	Male	3.68	-
PA79	Prelabs, LLC	Male	3.68	-

Supplementary Table 1: Demographics of Mauritian cynomolgus macaques used in the study.

Supplementary data 1: List of genes with fold change from the dd CyCMV/SIV IL-15 protection signature shown in Figure 3B. This is provided as a separate excel file.

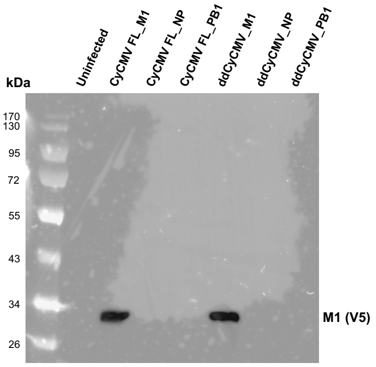

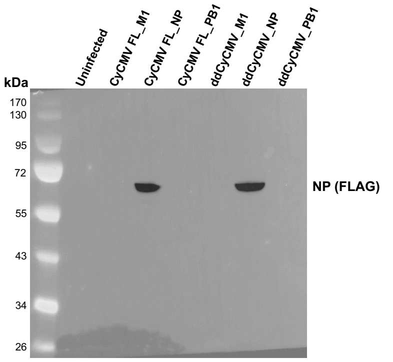

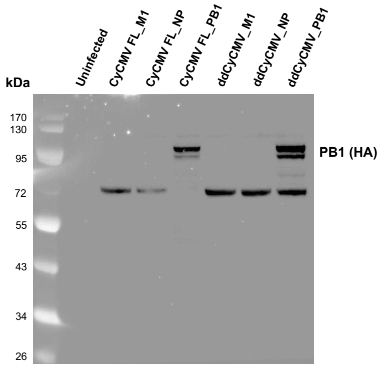

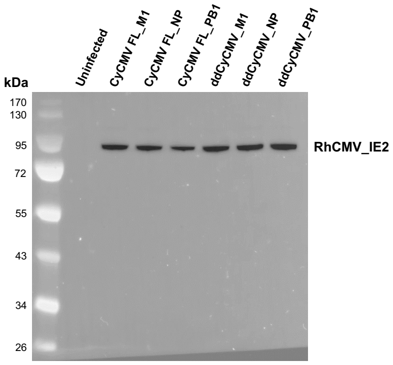

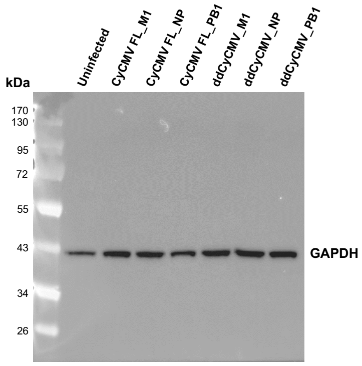

Source data: Uncropped immunoblots shown in Supplementary figure 1.
