# Supplementary material for: Cytomegalovirus vaccine vector-induced effector memory CD4 + T cells protect cynomolgus macaques from lethal aerosolized heterologous avian influenza challenge
Source: Nat Commun. 2024 Jul 19;15:6007. doi: 10.1038/s41467-024-50345-6 (PMC11272155; doi:10.1038/s41467-024-50345-6)
Supplement: Supplementary file 3 — Description of Additional Supplementary Files [file 41467_2024_50345_MOESM3_ESM.pdf]

**Description of Additional Supplementary Files**

**File Name: Supplementary Data 1**
**Description:** List of genes with fold change from the dd CyCMV/SIV IL-15 protection signature shown in Figure 3B.
