# Supplementary material for: Cytomegalovirus vaccine vector-induced effector memory CD4 + T cells protect cynomolgus macaques from lethal aerosolized heterologous avian influenza challenge
Source: Nat Commun. 2024 Jul 19;15:6007. doi: 10.1038/s41467-024-50345-6 (PMC11272155; doi:10.1038/s41467-024-50345-6)
Supplement: Supplementary file 5 — Reporting Summary [file 41467_2024_50345_MOESM5_ESM.pdf]

nature portfolio

Corresponding author(s): Simon Barratt-Boyes, Douglas S. Reed, and Jonah B. Sacha

Last updated by author(s): 5.14.2024

# Reporting Summary

Nature Portfolio wishes to improve the reproducibility of the work that we publish. This form provides structure for consistency and transparency in reporting. For further information on Nature Portfolio policies, see our Editorial Policies and the Editorial Policy Checklist.

## Statistics

For all statistical analyses, confirm that the following items are present in the figure legend, table legend, main text, or Methods section.

| n/a | Confirmed | |
|---|---|---|
| x | ☐ | The exact sample size (*n*) for each experimental group/condition, given as a discrete number and unit of measurement |
| x | ☐ | A statement on whether measurements were taken from distinct samples or whether the same sample was measured repeatedly |
| x | ☐ | The statistical test(s) used AND whether they are one- or two-sided<br>*Only common tests should be described solely by name; describe more complex techniques in the Methods section.* |
| x | ☐ | A description of all covariates tested |
| x | ☐ | A description of any assumptions or corrections, such as tests of normality and adjustment for multiple comparisons |
| x | ☐ | A full description of the statistical parameters including central tendency (e.g. means) or other basic estimates (e.g. regression coefficient) AND variation (e.g. standard deviation) or associated estimates of uncertainty (e.g. confidence intervals) |
| x | ☐ | For null hypothesis testing, the test statistic (e.g. *F*, *t*, *r*) with confidence intervals, effect sizes, degrees of freedom and *P* value noted<br>*Give P values as exact values whenever suitable.* |
| x | ☐ | For Bayesian analysis, information on the choice of priors and Markov chain Monte Carlo settings |
| x | ☐ | For hierarchical and complex designs, identification of the appropriate level for tests and full reporting of outcomes |
| x | ☐ | Estimates of effect sizes (e.g. Cohen's *d*, Pearson's *r*), indicating how they were calculated |

*Our web collection on statistics for biologists contains articles on many of the points above.*

## Software and code

Policy information about availability of computer code

| | |
|---|---|
| Data collection | Data collected using Ponemah v5.4 software was exported as 15-minute averages into Excel files. Flow data was collected on a BD Symphony using FlowJo 10.8.1. |
| Data analysis | Data from Ponemah were analyzed with Biaera software or MatLab 2019a. Flow data was analyzed using FlowJo 10.8.1. Images were processed and analysd with ImageJ software. Statistical analyses were done with SAS9.4. Nucleotide sequences were aligned using Clustal Omega,  followed by neighbor-joining phylogenetic tree construction using Geneious software 2022.2.2. The following softwares were used for RNAseq analyses: bowtie2 v2.4.2, STAR v2.7.5, bcl2fastq,  Gene counts were imported into the R statistical software for subsequent analyses. Differential expression analyses were performed with limma and EdgeR. |

For manuscripts utilizing custom algorithms or software that are central to the research but not yet described in published literature, software must be made available to editors and reviewers. We strongly encourage code deposition in a community repository (e.g. GitHub). See the Nature Portfolio guidelines for submitting code & software for further information.

## Data

Policy information about availability of data

All manuscripts must include a data availability statement. This statement should provide the following information, where applicable:

- Accession codes, unique identifiers, or web links for publicly available datasets
- A description of any restrictions on data availability
- For clinical datasets or third party data, please ensure that the statement adheres to our policy

All data reported in this paper will be shared by the lead contact upon request. Any additional information required to reanalyze the data reported in this paper is available from the lead contact upon request.

## Research involving human participants, their data, or biological material

Policy information about studies with human participants or human data. See also policy information about sex, gender (identity/presentation), and sexual orientation and race, ethnicity and racism.

| | |
|---|---|
| Reporting on sex and gender | *Use the terms sex (biological attribute) and gender (shaped by social and cultural circumstances) carefully in order to avoid confusing both terms. Indicate if findings apply to only one sex or gender; describe whether sex and gender were considered in study design; whether sex and/or gender was determined based on self-reporting or assigned and methods used.*<br>*Provide in the source data disaggregated sex and gender data, where this information has been collected, and if consent has been obtained for sharing of individual-level data; provide overall numbers in this Reporting Summary. Please state if this information has not been collected.*<br>*Report sex- and gender-based analyses where performed, justify reasons for lack of sex- and gender-based analysis.* |
| Reporting on race, ethnicity, or other socially relevant groupings | *Please specify the socially constructed or socially relevant categorization variable(s) used in your manuscript and explain why they were used. Please note that such variables should not be used as proxies for other socially constructed/relevant variables (for example, race or ethnicity should not be used as a proxy for socioeconomic status).*<br>*Provide clear definitions of the relevant terms used, how they were provided (by the participants/respondents, the researchers, or third parties), and the method(s) used to classify people into the different categories (e.g. self-report, census or administrative data, social media data, etc.)*<br>*Please provide details about how you controlled for confounding variables in your analyses.* |
| Population characteristics | *Describe the covariate-relevant population characteristics of the human research participants (e.g. age, genotypic information, past and current diagnosis and treatment categories). If you filled out the behavioural & social sciences study design questions and have nothing to add here, write "See above."* |
| Recruitment | *Describe how participants were recruited. Outline any potential self-selection bias or other biases that may be present and how these are likely to impact results.* |
| Ethics oversight | *Identify the organization(s) that approved the study protocol.* |

Note that full information on the approval of the study protocol must also be provided in the manuscript.

# Field-specific reporting

Please select the one below that is the best fit for your research. If you are not sure, read the appropriate sections before making your selection.

☒ Life sciences ☐ Behavioural & social sciences ☐ Ecological, evolutionary & environmental sciences

For a reference copy of the document with all sections, see nature.com/documents/nr-reporting-summary-flat.pdf

# Life sciences study design

All studies must disclose on these points even when the disclosure is negative.

| | |
|---|---|
| Sample size | Sample sizes for experimental groups was chosen based on previous studies with CyCMV vectors (see Malouli et al. CH&M 2022). |
| Data exclusions | No data exclusion |
| Replication | In vitro ICS data was replicated across 2-3 experiments for each animal to confirm results by repeating antigen recognition experiments. The results were replicated successfully. |
| Randomization | Animals were randomized into groups based on sex. |
| Blinding | All study staff involved in the H5N1 challenge studies were blinded to the vaccine status of the animals until completion of the study. |

# Reporting for specific materials, systems and methods

We require information from authors about some types of materials, experimental systems and methods used in many studies. Here, indicate whether each material, system or method listed is relevant to your study. If you are not sure if a list item applies to your research, read the appropriate section before selecting a response.

## Materials & experimental systems

| n/a | Involved in the study |
|---|---|
| ☐ | ☒ Antibodies |
| ☒ | ☐ Eukaryotic cell lines |
| ☒ | ☐ Palaeontology and archaeology |
| ☐ | ☒ Animals and other organisms |
| ☒ | ☐ Clinical data |
| ☒ | ☐ Dual use research of concern |
| ☒ | ☐ Plants |

## Methods

| n/a | Involved in the study |
|---|---|
| ☒ | ☐ ChIP-seq |
| ☐ | ☒ Flow cytometry |
| ☒ | ☐ MRI-based neuroimaging |

## Antibodies

Antibodies used

Antibody Clone ,Fluor, Supplier, Catalog #, Lot # ,Concentration (ng/T):
CD3, SP34-2, BUV395, BD Biosciences, 624310, 2294924, 120
CD8, SK-1, BUV737, BD Biosciences, 624235, 1354419, 50
CCR7, G043H7, Biotin, BioLegend, 93747, B376569, 200
Streptavidin, -- BV421, BioLegend ,92067, B343645, 150
HLA-DR, L243, BV510, BioLegend, 93784, B341690, 65
CD4, L200, BV786, Fisher Scientific, BDB565311, 2059603, 5
Ki67, B57, FITC, BD Biosciences, 624046, 2294951, 140,
CD95, DX2, PE, BioLegend, 94203, 110921, 27
CD28, CD28.2, PE/Dazzle 594, BioLegend, 93364, B287514, 40
CD69, CH/4, PE-Cy5.5, Life Technologies, MHCD6918, 2433447, 500
CCR5, 3A9, APC, BD Biosciences, 624346, 2277504, 80
CD20, 2H7, APC-Fire 750, BioLegend, 93924, B337142, 30
CD28, CD28.2, Pure, Life Technologies, CUST03277, C12015, 500
CD49d, 9F10, Pure, Life Technologies, CUST03278, C12016, 500
CD3, SP34-2, Pacific Blue, BD Biosciences, 624034, 9247857, 100
CD4, L200, BV510, BD Biosciences, 624340, 7128982, 220
TNFα, Mab11, PE, BioLegend, 96019, B379985, 20
TNFα, Mab11, FITC, BioLegend, 624046, 2294951, 140
CD69, FN50, PE, eBiosciences, CUST01282, 60
CD69, FN50 PE/Dazzle 594, BioLegend, 93437, B267627, 50
CD8α, SK-1, PerCP-eFluor L710, Life Technologies, CUST04424, C11972, 8
IFNγ, B27, APC, BioLegend, 96018, B274243, 80

Validation

Antibody cross-reactivity is listed at the NHP Reagent Resource website: https://www.nhpreagents.org

## Animals and other research organisms

Policy information about studies involving animals; ARRIVE guidelines recommended for reporting animal research, and Sex and Gender in Research

Laboratory animals

Mauritian-origin cynomolgus macaques (MCM) were purchased from Prelabs following serological testing confirming the absence of antibodies to influenza A and B viruses. Specifics information for each animal is listed in supplemental table 1.

Wild animals

N/A

Reporting on sex

MCM were split randomly into three experimental groups based on sex to yield equal numbers of male and female MCM in each group.

Field-collected samples

N/A

Ethics oversight

Animal Care and Use Committee of Tulane University. University of Pittsburgh, which is fully accredited by the Association for Assessment and Accreditation of Laboratory Animal Care International. Prior to challenge, the work with animals described in this report was approved by the University of Pittsburgh's Institutional Animal Care & Use Committee (IACUC).

Note that full information on the approval of the study protocol must also be provided in the manuscript.

## Plants

Seed stocks

*Report on the source of all seed stocks or other plant material used. If applicable, state the seed stock centre and catalogue number. If plant specimens were collected from the field, describe the collection location, date and sampling procedures.*

Novel plant genotypes

*Describe the methods by which all novel plant genotypes were produced. This includes those generated by transgenic approaches, gene editing, chemical/radiation-based mutagenesis and hybridization. For transgenic lines, describe the transformation method, the number of independent lines analyzed and the generation upon which experiments were performed. For gene-edited lines, describe the editor used, the endogenous sequence targeted for editing, the targeting guide RNA sequence (if applicable) and how the editor was applied.*

Authentication

*Describe any authentication procedures for each seed stock used or novel genotype generated. Describe any experiments used to assess the effect of a mutation and, where applicable, how potential secondary effects (e.g. second site T-DNA insertions, mosiacism, off-target gene editing) were examined.*

## Flow Cytometry

### Plots

Confirm that:

☒ The axis labels state the marker and fluorochrome used (e.g. CD4-FITC).

☒ The axis scales are clearly visible. Include numbers along axes only for bottom left plot of group (a 'group' is an analysis of identical markers).

☒ All plots are contour plots with outliers or pseudocolor plots.

☒ A numerical value for number of cells or percentage (with statistics) is provided.

### Methodology

Sample preparation: PBMC was isolated from EDTA-treated whole blood.

Instrument: LSRII (BD Biosciences)

Software: FlowJo

Cell population abundance: No cell sorting was performed

Gating strategy: See supplemental figure 2

☒ Tick this box to confirm that a figure exemplifying the gating strategy is provided in the Supplementary Information.
